#  Synthetic Genomics and Synthetic Biology Applications Between Hopes and Concerns

**DOI:** 10.2174/1389202911314010003

**Published:** 2013-03

**Authors:** Harald König, Daniel Frank, Reinhard Heil, Christopher Coenen

**Affiliations:** 1 Institute for Technology Assessment and Systems Analysis (ITAS); 2 Institute of Toxicology and Genetics (ITG), Karlsruhe Institute of Technology, PO box 3640, 76021 Karlsruhe, Germany

**Keywords:** Applications, Benefits, Biofuels, Biomedicine, Environment, Risks, Synthetic genomics, Synthetic biology.

## Abstract

New organisms and biological systems designed to satisfy human needs are among the aims of synthetic genomics and synthetic biology. Synthetic biology seeks to model and construct biological components, functions and organisms that do not exist in nature or to redesign existing biological systems to perform new functions. Synthetic genomics, on the other hand, encompasses technologies for the generation of chemically-synthesized whole genomes or larger parts of genomes, allowing to simultaneously engineer a myriad of changes to the genetic material of organisms. Engineering complex functions or new organisms in synthetic biology are thus progressively becoming dependent on and converging with synthetic genomics. While applications from both areas have been predicted to offer great benefits by making possible new drugs, renewable chemicals or clean energy, they have also given rise to concerns about new safety, environmental and socio-economic risks – stirring an increasingly polarizing debate. Here we intend to provide an overview on recent progress in biomedical and biotechnological applications of synthetic genomics and synthetic biology as well as on arguments and evidence related to their possible benefits, risks and governance implications.

## INTRODUCTION

 Giving an unequivocal definition of synthetic biology is challenging, even to the various actors in the field ([1-3] and references therein). Rather than constituting a strictly defined field, synthetic biology may be best described as an engineering-related approach to rationally design and construct biological compounds, functions and organisms not found in nature, or to redesign existing biological parts and systems to carry out new functions. It integrates different scientific disciplines, including molecular and systems biology, chemistry, (bio-)physics, computer-aided modeling and design as well as an engineering-based notion of generating and using interchangeable “biological parts” (such as regulatory DNA and RNA elements, or coding sequences for proteins/protein domains) [1-4]. Compared to “traditional” genetic engineering, which mostly enhances existing biological functions or transfers them between organisms based on the modification or transfer of one or very few genes, synthetic biology work may be characterized as involving the combination of multiple genes, newly constructed “biological parts” or the use of non-natural molecules to enhance traits or to construct new biological pathways and functions – and (in the future) entire organisms. Furthermore, rational design processes are increasingly guided by *in silico* modeling. However, we would like to note that some work from genetic engineering and molecular biology from the last 20-25 years overlaps with today’s synthetic biology concepts of generating new biological parts and systems with new functions. Examples of this are reporter gene systems to indicate water and soil contaminants [5] or gene expression pattern in organisms [6], as well as conditional gene expression systems for mammalian cells controlled by antibiotics such as tetracycline [7]. Who would deny that they were generated based on the rational combination of “biological parts” with known function (i.e. regulatory DNA-elements and DNA sequences encoding proteins/protein domains) or even by constructing new “parts” (if we think of the hybrid transcriptional regulators made of viral and bacterial protein domains that confer tetracycline-control to mammalian cells [7]) – and that they have generated new functions? A similar overlap and coupling to synthetic biology has been recently proposed by Nielsen and Kiesling [8] for metabolic engineering (the metabolic analysis and genetic engineering of cells for improving an designing metabolic pathways). Between traditional approaches, that only increase the pathway flux towards a desired product by directed genetic modifications in a naturally producing strain, and synthetic biology’s envisioned generation of complete synthetic cells designed to produce the desired product, there would be approaches that use concepts of both traditional metabolic engineering and synthetic biology. These would involve using cells that normally do not produce a product of interest, but which are able to do so after being equipped with a “synthetic” pathway – though initially often in small amounts only. In a second step, the flux through the pathway is then increased by traditional metabolic engineering [8].

 Synthetic genomics has been defined as the engineering and manipulation of an organism’s genetic material on the scale of the whole genome, based on technologies to design and chemically synthesize pieces of DNA and to assemble them to long, chromosome-sized fragments [9, 10]. These can serve as entire genomes of viruses or bacteria [11, 12]. Compared with traditional genetic engineering, where typically only very few nucleotides or genes in an organism are altered (mostly based on recombinant DNA technology), synthetic genomics thus allows to simultaneously change a large number of nucleotides or gene loci all over the genome by gene synthesis. 

 Since synthetic biology aims to engineer complex biological features and to effectively integrate them into organisms as well as to construct entire, new organisms, the field may increasingly integrate, require and converge with synthetic genomics [10-12]. In fact, approaches to apply synthetic biology ideas have begun to go far beyond first combinations of very few natural “parts”, for example, to build reporter genes responsive to heavy-metal ions [5]. Increasingly complex gene circuits have been generated, such as those used to detect multiple changes in cancer cells [13], or computer-modeled, sophisticated non-natural metabolic pathways to produce chemicals and fuels have been constructed [14, 15]. Furthermore, synthetic genomics techniques have been used to reconstruct viruses including polio virus or the virus of the 1918 influenza pandemic [12], to introduce genome-wide changes for designing vaccine candidates from the poliovirus and influenza viruses [12, 16], or to generate a first bacterial (*Mycoplasma*) cell controlled by a chemically synthesized genome upon transplantation into a related recipient cell [11]. 

 Besides giving us greater knowledge about how living things operate and how life could originally have emerged, societal benefits derived from synthetic genomics/biology applications such as novel drugs and vaccines or “greener” chemicals and biofuels for climate-change mitigation have been proposed – all of which may contribute to a new revolutionary bioeconomy [17-19]. Conversely, potential biosafety, biosecurity [9, 20, 21], environmental and socio-economic risks [21-23], as well as ethical and other philosophical concerns regarding the nature of life [24] have been raised. Against this background, we wish to provide a sober, evidence-based picture of the current state of synthetic genomics/biology applications and of their potential benefits and risks in economically and politically relevant areas, namely health, the environment and energy.

## HEALTH

**Fig. (1):**
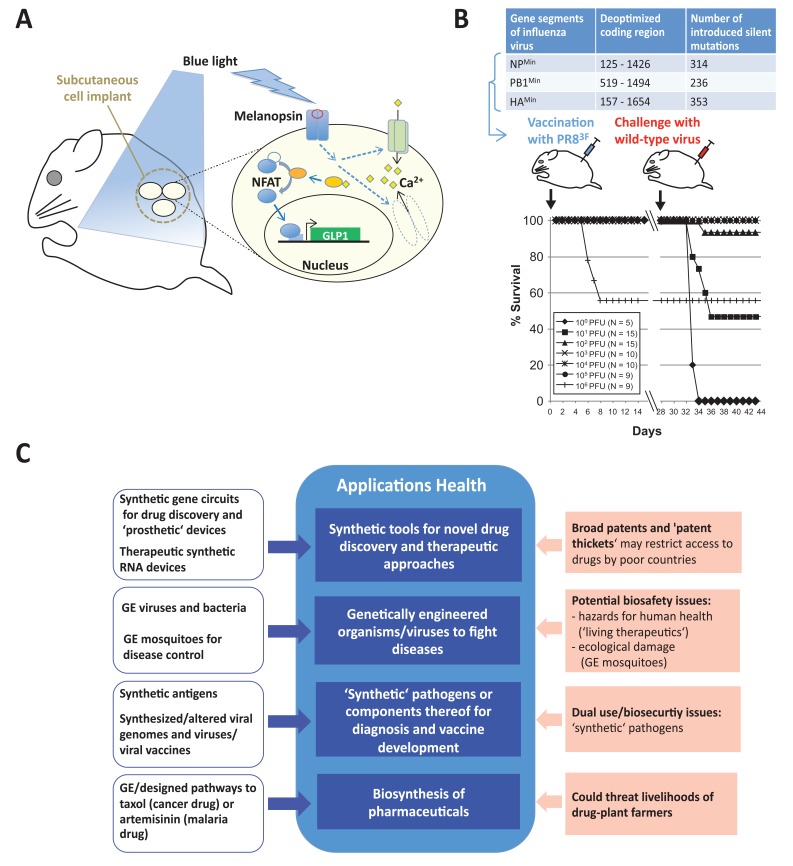
Synthetic genomics and synthetic biology applications in biomedicine and health. **A.** “Prosthetic” cell implants to control glucose levels by blue light in a mouse model of human type-2 diabetes [32]. Implants contain encapsulated human cells (yellow ovals) with an assembled signal transduction pathway that couples light detection by melanopsin (a blue-light receptor from the retina) via calcium (Ca^2+^) signaling to activation of a transcriptional gene activator, the nuclear factor of activated T cells (NFAT). NFAT in turn activates expression of glucagon-like peptide 1 (GLP1), a type-2 diabetes drug candidate, from a gene construct controlled by NFAT DNA-binding elements. **B.** Generation of live attenuated influenza virus vaccines by synthetic attenuated virus engineering (SAVE) [16] (adapted/reprinted with the permission of Macmillan Publishers Ltd: Nature Biotechnology, Mueller *et al*., Nat Biotechnol 28, 723-726, copyright 2010). Hundreds of nucleotide changes were introduced into three genes of the influenza virus (NP, PB1, and HA; see top panel) by computer-aided rational design and gene synthesis. While preserving the amino acid sequence of the encoded proteins, these changes rearrange existing synonymous codon pairs and reduce protein synthesis. A synthetic virus containing the three deoptimized genes (PR8^3F^) can be safely used to immunize mice: they survived vaccine administration with this attenuated virus, except in the case of very high doses (bottom panel, at left); and safe vaccine doses led to immune protection following a lethal dose of the wild-type virus (bottom panel, at right). **C.** Overview of synthetic genomics and synthetic biology approaches in biomedicine and health (open blue boxes) and of their potential applications/assets (filled blue boxes) and challenges/risks (filled red boxes). GE, genetically engineered.

 While chronic non-communicable diseases (including cardiovascular disorders, cancer or diabetes) are the major causes of death in rich-world countries and are reaching substantial proportions worldwide, most of the major challenges facing the developing world have been defined in vaccine development and fighting infectious diseases [25, 26]. Various synthetic genomics/biology-related approaches (for an overview, see Fig. **1C**) address the health challenges faced by both types of countries.

### (i) Synthetic Circuits and Devices for Drug Discovery and Therapeutic Applications

 Simple synthetic transcription circuitries involving hybrid transcription regulators, constructed by combining bacterial and viral protein domains, have been developed that allow to screen potential drugs for tuberculosis [27] or HIV infection [28] in a mammalian cell line or bacteria, respectively. In contrast, more complex or multi-input sensing, gene-expression regulatory circuits and synthetic RNA molecules have been designed that can detect disease-associated molecular changes in cells (e.g. cancer cells) and activate cell-death pathways to eliminate the diseased cells [13, 29, 30]. Furthermore, “prosthetic devices” can be built consisting of transplantable, microencapsulated mammalian cells equipped by synthetic gene circuits or assembled signaling pathways. Such devices have been used in mouse disease models to restore urate homeostasis using a chimeric urate sensor system to control secretion of an engineered fungal urate oxidase [31], or for the blue light-induced (optogenetic) control of glucose levels [32] (for details see Fig. **1A**). Such approaches may offer efficient new ways for drug screening via designed disease-specific pathways *in vitro* (i.e. outside the body) or for therapies based on transplantation of therapeutic devices. Yet, strategies based on synthetic small RNA devices may suffer from still unsolved issues of systemic or targeted delivery, if applications depend on delivery to cells in the organism [33]. 

### (ii) Genetically Engineered Viruses and Organisms to Fight Disease

 A combination of classical genetic engineering (through modification or transfer of one or two natural genes) and the synthetic biology concept of rationally designing functions not present in nature has been employed to reengineer viruses that specifically kill cancer cells. Thus, an adenovirus was constructed in which viral genes needed for its replication (E1A and E4) were brought under the control of the gene regulatory region of the human E2F1 gene that in normal cells is repressed by the RB tumor suppressor gene product. Since many tumor cells lost this suppressor, selective viral gene expression, replication, and progeny production/cell lysis can occur in a variety of tumor cells, but not normal cells [34]. Similarly, a pox virus that shows tumor-specific replication (in clinical trials) based on targeting multiple mechanisms [35] was rationally constructed by simple genetic engineering, involving viral thymidine kinase gene inactivation and expression of a human transgene for granulocyte-macrophage colony stimulating factor (GM-CSF) [36]. 

 Further approaches for targeting cancer cells have been devised to equip bacteria with multiple functions, based on introducing various heterologous genes as well as synthetic gene constructs. So the ability to invade human cancer-derived cells was conferred to *E. coli *through bringing in genes for invasins from other bacterial species; in one case this function was made dependent on cell density and hypoxic conditions typical for tumors, by coupling invasin expression to corresponding synthetic sensor modules in *in-vitro* experiments [37]. In another case, invasin expression was combined with the expression of the listeriolysin O gene *HlyA *and a synthetic gene construct to generate short hairpin RNAs (shRNAs) interfering with expression of a tumor cell factor. This allowed shRNA delivery from *E. coli* to normal mammalian cells as well as tumor cells, both in cell culture and in mice [38].

 Finally simple genetic circuits, however based on chimeric regulatory-proteins or synthetic genetic elements, have been devised to eradicate insect vector populations for parasites causing malaria or dengue fever. Thus simple tetracycline-repressible genetic circuits introduced to *Aedes aegypti* (the mosquito that spreads dengue fever) produce lethality in the offspring, in the absence of tetracycline, when genetically-engineered mosquitoes mate with wild ones. Several of these systems are designed to generate female-specific lethality [39, 40]. In a different strategy, synthetic mobile genetic elements that can invade mosquito populations (as recently shown for human malaria mosquitoes [41]) could provide a tool capable of rapidly spreading genetically-engineered parasite resistance among mosquitoes in the field [41].

### (iii) Synthesis of Pathogens or Components Thereof for Diagnosis and Vaccine Development

The generation of chimeric antigens exemplifies a relatively straightforward approach to construct novel diagnosis tools for pathogens (e.g. Lyme disease) involving DNA-synthesis [42]. Rather complex DNA-synthesis and genome-assembly techniques have been used to generate entire viral genomes and to address the etiology and pathogenicity mechanisms of corresponding viruses, including the viruses that caused the 1918 influenza pandemic or that are responsible for SARS [43, 44]. Similarly, such synthetic genomics technology can be exploited to introduce hundreds of base-pair changes in codon pairs in order to produce live attenuated viral vaccines by means of computer-aided rational design. This approach has been demonstrated to rapidly generate safe and effective influenza vaccine candidates in mice [16] (Fig. **1B**).

### (iv) Biosynthesis of Pharmaceutically Active Compounds

 The production of naturally occurring drugs (especially the anti-malaria compound artemisinin) through assembled complex metabolic pathways in microorganisms has become one of the best-known applications linked to synthetic biology [2, 3]. Crucial precursors for plant-derived drugs like artemisinin, which is effective against multi-resistant forms of malaria in combination therapy [45], or for one of the most important cancer drugs, taxol (paclitaxel) [46], can so be produced in yeast and *E. coli*, respectively. In both cases, new pathways leading to the desired products were assembled from a native upstream part, (involving yeast and *E. coli* enzymes, respectively) and a heterologous downstream part composed of plant enzymes. The flux through these composite pathways was increased mainly by upregulation of several rate-limiting pathway components. Both approaches thus apply concepts from “traditional” metabolic engineering and synthetic biology [8]. 

 However, also synthetic biology approaches including non-natural amino acids and expanded genetic codes have been envisaged for the biosynthesis (see, e.g. [47]) and the diversification of peptide-based compound libraries [48] in bacteria, from which drugs can be selected by functional screening approaches (see, e.g. [28]). 

## ENVIRONMENT

**Fig. (2):**
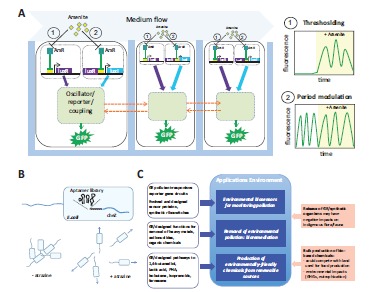
Synthetic biology approaches to environmental applications. **A.** Whole-cell biosensor array that is frequency-modulated by arsenite [55]. The array consists of multiple *E. coli* colonies (“biopixels”; schematically represented by single cells, black rounded rectangles) growing in wells of a microfluidic device (light blue), through which media can be pumped (top). Cells contain an oscillator module based on genetic quorum-sensing circuits (light green), producing synchronized oscillations of the expression of H_2_O_2_ and of green fluorescent protein (GFP). H_2_O_2_ can migrate between colonies and synchronize them by affecting the oscillator module. This genetic oscillator was coupled to one of two arsenite sensor modules (1 or 2) containing parts of the oscillator (luxR or luxl genes) under the control of an arsenite-responsive repressor protein (ArsR) and its cognate promoter element (yellow box). Oscillation can thus either be switched on/off (thresholding, 1) or modulated in frequency (period modulation, 2) by arsenite and measured via GFP fluorescence (panels on the right). **B**. Generation of synthetic riboswitches to generate bacteria that detect, follow and can destroy the herbicide atrazine [53]. A library of atrazine-binding small RNAs (aptamers) selected *in vitro* was inserted into the 5'-untranslated region of the *cheZ* gene that controls *E. coli* motility, upstream of a randomized sequence (NNN, upper panel). Together with an aptamer, the randomized sequence can become part of a riboswitch element that couples ligand binding and translational control of *cheZ* mRNA. By functional screening, riboswitches were selected that mediated atrazine-dependent cell motility (bottom panel). When an atrazine-degrading enzyme is introduced, the cells migrate towards atrazine and degrade it. **C.** Overview of approaches related to synthetic biology addressing environmental issues (open blue boxes) and of their potential applications/ assets (filled blue boxes) and associated challenges/risks (filled red boxes). GE, genetically engineered; GHGs, greenhouse gases; PHA, polyhydroxy-alkanoates.

 The toxic contamination of soil and water and an increase in atmospheric greenhouse gases (GHGs) due to human activities, including industrial production processes and the use of fossil fuels, have become major environmental issues on a global scale [49]. These may be addressed by several approaches related to the synthetic biology idea (summarized in Fig. **2C**).

### (i) Environmental (Whole-Cell) Biosensors

 Bacteria equipped with pollutant-responsive gene-regulation units and/or metabolic pathways for pollutants, coupled to reporter genes, have early been generated to detect and measure environmental contaminants such as heavy metals, explosives or pesticides. The introduced genetic circuits can vary greatly in complexity, however, and examples comprising natural sensor proteins [5, 50] can be distinguished from those that involve “non-natural” sensor proteins with novel specificities, generated by directed evolution [51] or computational design [52]. Though involving few components, the approaches involving “non-natural” sensor proteins as well as the recent integration of a synthetic riboswitch capable of controlling migration of green fluorescent protein expressing bacteria towards the pesticide atrazine (followed by its degradation) [53] (see Fig. **2B**) can exemplify the overlap of these rather simple genetic engineering approaches with synthetic biology concepts of (re-)designed biological parts and the generation of functions not found in nature. Despite technical challenges such as the maintenance of viability/activity, reporter noise and pollutant specificity [54], whole-cell biosensors can enable cheap and simple large-scale field measurements of collected samples [5].

 Interestingly, a recent more sophisticated approach guided by computational modeling combines synthetic arsenite-sensing gene circuits with an oscillating circuit in *E. coli*, allowing heavy-metal-dependent frequency modulation of fluorescence signals with a large dynamic range. At the same time, a large number of these fluorescent biosensor cell colonies were coupled and synchronized via a rapidly diffusible, long-range output signal (hydrogen peroxide) generated by the synthetic circuits [55] (Fig. **2A**). Such synchronization and integration of signals from millions of cells could prove generally applicable to overcome an important issue in the construction of robust circuits, namely the considerable intercellular variability in circuit behavior due to noisy processes. These include random bursts of transcription and translation, or differences in the growth state of individual cells (see [54, 55] and references therein).

### (ii) Removal of Environmental Pollution by Genetically Engineered Organisms

 While decontamination of water or soil by microorganisms (bioremediation) is a process that can occur naturally (intrinsic bioremediation), it may be enhanced by the addition of nutrients (biostimulation), additional microorganisms (bioaugmentation) or by plants (phytoremediation) [56].

Approaches adopting synthetic biology principles include the construction of hybrid proteins combining bacterial and mammalian protein domains, or proteins generated by directed evolution that upon transfer in bacteria provide new or enhanced functions in binding or reducing heavy metal ions and radionuclides [57, 58]. Of note, approaches to generate bacteria for biodegradation of organic chemicals took on the synthetic biology concept to build new biochemical pathways from various “bioparts” already in the late 1980s. Thus, a novel and complex catabolic pathway for the degradation of the organic pollutants methylphenols and methylbenzoates have been constructed in *Pseudomona* sp. by combining “in patchwork fashion“ [59] enzymes from five different catabolic pathways of three distinct soil bacteria [59]. Similarly, though somewhat less complex, a new pathway for biodegradation of 2-chlorotoluene was assembled from segments of distinct catabolic pathways [60]. 

 More recently the function to seek and degrade a pollutant was generated by coupling in *E. coli* a synthetic aptamer riboswitch that recognizes the herbizide atrazine to the translation of the mRNA for the protein (CheZ) controlling *E. coli* motility. Additionally, these cells were equipped with an atrazine chlorohydrolase gene (*atz*A) for atrazine degradation, derived from *Pseudomonas* sp. soil bacteria [53] (Fig. **2B**).

### (iii) Production of Chemicals from Renewable Sources

 Based on multiple genes from different organisms, pathways have been constructed and optimized by metabolic engineering [8] to efficiently produce chemicals in microorganisms and plants. For instance, in an attempt to produce biodegradable plastics, a pathway to synthesize lactic acid from sugar was generated in *E. coli* [61] and switchgrass (*Panicum virgatum* L.) was engineered to produce polyhydroxy-alkanoates (PHA) [62]. Likewise, a complex pathway (made of an optimized plant gene and 8 genes from yeast) was transferred to and expanded in *E. coli* to synthesize isopenoids [63], and various genes from *Klebsiella pneumonia* and yeast were used to construct a pathway in *E. coli* to directly produce the commodity chemical 1,3-propanediol from glucose [64]*. *Interestingly, a new efficient pathway for the biosynthesis of a chemical that is produced in nature in trace amounts only was constructed by adding a single enzyme activity to an organism. Thus, an artificial pathway for the efficient production of isobutene, a chemical that can be used to synthesize plastics, rubber or fuels, has been generated in *E. coli*, based on the introduction of a mevalonate diphosphate decarboxylase (MDD) activity derived from a protein-engineered version of an MDD from an archebacterium (that cannot produces isobutene) [65]. 

 Finally, we would like to acknowledge the recent generation of *E. coli* strains that directly produce 1,4-butanediol (BDO) [66], an important commodity chemical for products such as plastics, rubber or solvents. This example is especially noteworthy since (compared to the previous examples) it has moved metabolic pathway engineering closer towards a central synthetic biology concept, namely the application of rational computer-based design and modeling to generate biological functions not present in nature (or, in future, to build entire new organisms). BDO is a non-natural chemical not produced by any known organism; its production thus required the construction of a new pathway with no “blueprint” in nature. Moreover, this new pathway was constructed involving a high degree of rational design based on *in silico* algorithms predicting and ranking possible pathways from *E. coli* central metabolites to BDO (involving both native *E. coli* and heterologous enzyme activities). This was followed by optimizing the strain to channel carbon and energy sources into the new pathway via gene deletions guided by an *E. coli* genome-scale metabolic model ([66] and references therein).

## ENERGY/BIOFUELS

**Fig. (3):**
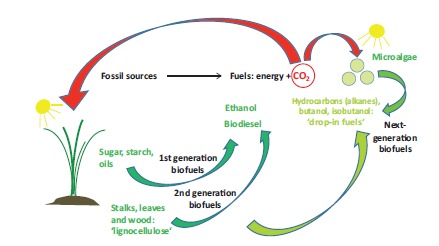
Different generations of biofuels and their carbon cycles.

**Fig. (4):**
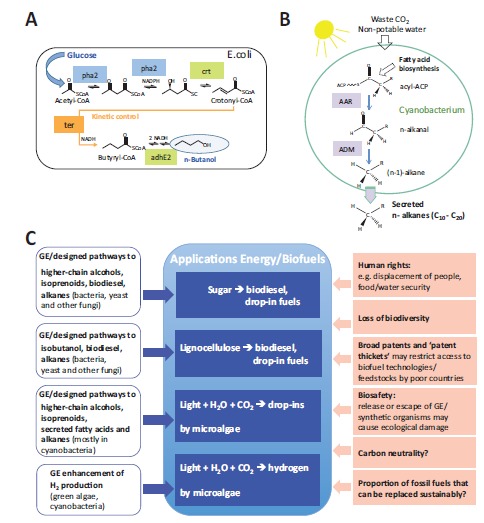
Approaches involving synthetic biology concepts to generate biofuels. **A**. A synthetic pathway for high-level production of *n*-butanol from glucose in *E. coli* involving six enzymatic reaction steps based on genes from different organisms [14]. It includes the generation of an enzymatic reaction mechanism (rather than a physical one) as a kinetic control element to achieve high yields (shown in orange). *phaA* acetoacetyl- CoA thiolase/synthase; *phaB*, 3-hydroxybutyryl-CoA dehydrogenase; *crt*, crotonase; *ter*, trans-enoyl-CoA reductase; *adhE2* bifunctional butyraldehyde and butanol dehydrogenase. The origin of genes is indicated by color: blue, *R*. *eutropha*; green, C. *acetobutylicum*; orange, *T. denticola*. **B**. Generation of cyanobacteria for direct photosynthetic production and secretion of hydrocarbon fuels (n-alkanes) involving waste CO_2_ and non-potable water [17-86] The introduction of as few as two heterologous genes, encoding acyl-ACP reductase (AAR) and alkanal decarboxylative mono-oxygenase (ADM) activities, from other cyanobacterial species can confer, or enhance, n-alkane (C_10_- C_20_) synthesis (dependent on the host cell) and directs product secretion for easy recovery without the need to extract cells [86]. **C**. Overview of approaches related to synthetic biology (open blue boxes) and of their potential applications/assets (filled blue boxes) and challenges/risks (filled red boxes) in the production of biofuels. GE, genetically engineered.

 In an attempt to create renewable and sustainable carbon-based fuels, first-generation biofuels have been developed that are based on plant oils (biodiesel) or on cane sugar and crop starch (ethanol); see Fig. (**3**). Besides the “fuel-vs.-food” issue and negative effects on GHG emissions and biodiversity from land-use change (see below), these fuels have undesirable chemical properties that prohibit their use for certain purposes or with existing infrastructure [67]. New generations of biofuels based on non-edible, lignocellulosic plant parts, special energy grasses or microalgae have thus been envisaged [17, 68-70] (Fig. **3**). In addition, rather than producing biodiesel or ethanol, some approaches aim to create “drop-in” fuels that can use existing infrastructure and can be mixed with fossil fuels in any ratio. These are based on synthetic hydrocarbons or higher-chain alcohols (like butanol) with high energy content, allowing gasoline, diesel and even aviation fuels to be replaced [67, 71]. In addition, strategies have been devised that use microorganisms to produce hydrogen [72]. All these approaches involve synthetic biology ideas and can be ascribed to one of two fundamental strategies: the microbial synthesis of fuels from materials produced by plants (see (i) and (ii), below) or their direct microbial photosynthesis from CO_2_ and water ((iii) and (iv)) (see also overview in Fig. **4C**). 

### (i) Sugar to Biodiesel and Drop-In Fuels

 The transfer of multiple genes from different organisms into *E. coli*, and the deletion of different endogenous genes, allowed efficient new pathways to be generated for producing butanol [14, 73, 74] and branched-chain higher alcohols [73] from sugar. Of special interest with regard to the synthetic biology idea is that these include synthetic pathways based on computer-aided design with enzyme-based kinetic control mechanisms, allowing the efficient production of the non-native alcohol products [14, 74] (Fig. **4A**). The generation of new pathways based on combining different genes from distinct organisms also allowed biodiesel and alkanes to be synthesized from sugar in *E. coli*, yeast and other fungi [75-77], and alkanes in microalgae [76]. Noteworthy, for biodiesel production a first sensor-regulator system was designed and integrated in *E. coli* to adapt gene expression dependent on levels of a key intermediate (fatty acids) that significantly increased product yield [15]. 

 Since these approaches use available sugar (e.g. from sugar cane or corn), they do not depend on expensive technology to obtain sugar from lignocellulose or for direct light conversion (e.g. photobioreactors). At the same time, they allow to synthesize drop-in fuels (see above). 

### (ii) Lignocellulose to Biodiesel and Drop-In Fuels

 Several pathway engineering approaches aim to synthesize biofuels from lignocellulosic polysaccharides (cellulose, hemicellulose), making available non-edible plant parts. For example, a pathway to produce isobutanol was constructed in a native cellulose-degrading bacterium using various genes from different organisms [78]. Similarly, genes to make use of cellulose or hemicellulose were transferred into different microorganisms, some of them containing one or several exogenous genes, in order to synthesize butanol, biodiesel or hydrocarbons [77, 79, 80]. 

### (iii) Direct Synthesis of Biodiesel and Drop-In Fuels from Light, Water and CO_2_

 Higher photosynthetic yields per area, less need for arable land or the use of brackish or sea water [81] have inspired various approaches aimed at producing biofuels in microalgae. These include the generation of new metabolic pathways by combining genes from distinct organisms in cyanobacteria to synthesize products that can be converted to or act as drop-in fuels, such as isobutyraldehyde or butanol derivatives [82-84]. Cyanobacteria have also been metabolically engineered to efficiently produce and secrete fatty acids (to synthesize biodiesel) [85] or alkanes [86], with secretion allowing continuous and energy-saving production schemes [17]. In contrast to the previous examples, in the case of alkane synthesis and secretion, introducing as few as two genes from other cyanobacteria was sufficient to build a pathway for linear alkane synthesis and a module for alkane secretion, allowing to generate these functions in robust cyanobacterial genera that may be exploited for industrial use [86] (Fig. **4B**). However, although different studies have suggested the technical feasibility and economic viability of industrial-scale biofuel production by microalgae (e.g. [17, 87, 88]), there is as yet no such product on the market.

 Direct photosynthetic production of biofuels may strongly benefit from higher efficiencies in solar light conversion and by reducing the amount of biomass that has to be grown. A nascent concept taking these factors into account is microbial electrosynthesis, which may be described as an artificial form of photosynthesis to produce organic compounds and energy-rich fuels [89]. Here the higher efficiency in photovoltaics to harvest light energy (compared to natural photosynthesis) can be used to supply electrons via electrodes to certain microorganism in order to reduce CO_2_ to multicarbon products. Recently, electrosynthesis of isobutanol and 3-methyl-1-butanol (which can be used as drop-in fuels) has been achieved by introducing a synthetic metabolic pathway to these products (previously constructed in *E. coli* [73]) into the lithotrophic bacterium *Ralstonia eutropha* H16 [90].

### (iv) Microbial Synthesis of Hydrogen from Light and Water

 The prospective use of hydrogen as a non-carbon fuel has raised interest in certain photosynthetic microorganisms, such as algae and cyanobacteria, that can produce hydrogen from water and light [72]. Effective production, however, is limited by several issues, including the oxygen sensitivity of hydrogenases (the enzymes that can reduce protons and release molecular hydrogen) and inefficiencies in utilisation of solar light energy. So far, several approaches based on alteration of the expression of genes, including those for light-harvesting proteins, or the introduction of heterologous hydrogenase genes have aimed to improve hydrogen production by increasing the efficiency of light conversion or by reducing oxygen production or sensitivity in green algae [91, 92] and cyanobacteria [93, 94]. Rather than these more “traditional” genetic engineering approaches, synthetic biology approaches to construct new pathways for microbial hydrogen production might significantly contribute to solving these problems in the future.

## SOCIETAL BENEFITS AND CONCERNS

### Health

 With respect to public health (see overview in Fig. **1C**), new drug discovery and diagnosis tools to combat important infectious diseases, such as tuberculosis [27], may be expected. Likewise, novel therapeutic strategies based on “synthetic” viruses, organisms or engineered mammalian cells to fight cancer [13, 36, 37], important parasite diseases (like malaria or dengue fever) [40, 41] or metabolic disorders (such as diabetes or gout) [31, 32] may emerge. Furthermore, synthesizing drugs by metabolically-engineered microbes may provide more affordable alternatives to expensive chemical synthesis or extraction from precious natural sources, as in the case of the anti-cancer drug taxol [95]. Similarly, it may allow demand to be met for natural products that are in short supply and/or need a stable source, as in the case of artemisinin for malaria therapy [96]. Finally, viral vaccines (e.g. influenza vaccines) may become more easily and rapidly available [12, 16], and thus could help to prevent pandemics.

 Nonetheless, a number of challenges and concerns related to these approaches in the health area (see Fig. **1C**) may be worth considering. These include potential biosafety issues linked to the use of genetically engineered organisms as therapeutics. For example, bacterial toxicity or host immune response to bacteria and viruses may occur, or there may be genetic changes with unpredictable consequences during the proliferation cycle of “living therapeutics” such as viruses or bacteria [97, 98].

Similarly, while the strategy underlying the use of genetically-engineered mosquitoes to combat dengue fever [40, 99] may provide a species-specific means of controlling insect vectors for important diseases, various concerns have been pointed out. These include potential hazards for human health (by synthetic gene products injected by bites of surviving female mosquitoes), potential ecological consequences (e.g. empty niche) [100, 101] or ethical and social issues [101, 102]. Nonetheless, initial field-release experiments have recently been performed in the Cayman Islands, Malaysia, and in Brazil [99, 101]. Further socio-economic issues may arise from a soar in broad patents and “patent thickets” through synthetic biology [103, 104], potentially impeding access to drugs by poor countries. The production of plant-derived drugs by metabolically-engineered microbes could also threaten the livelihoods of drug-plant farmers in developing countries, a concern expressed early on in connection with synthetic biology-derived artemisinin [21, 105] which was supposed to be commercialised in 2012/early 2013 [106]. Finally, sequence information and knowledge about pathogen genomes, coupled with advanced and relatively cheap custom DNA synthesis and genome assembly, has raised biosecurity concerns regarding potential misuse (for a recent review, see [20]; and also below). 

### Environment

 Hopes in the field of the environment (see overview in Fig. **2C**) include biosensors for environmental toxins and bioremediation strategies based on genetically engineered microorganisms (GEMs). These applications have been proposed to allow efficient and cheap monitoring and removal of pollutants without destroying a site’s material, flora and fauna [107, 108]. Furthermore, an industry based on the environmentally-friendly and sustainable biosynthesis of chemicals and materials could reduce the depletion of and dependence on fossil resources and mitigate climate change. 

 These hopes have so far been countered above all by concerns about biosafety and sustainability (see Fig. **2C**). For example, *in situ* applications of GEMs for biosensing and bioremediation will require GEMs to be released into the environment and some experiments point to possible impacts on indigenous organisms through horizontal gene transfer of recombinant DNA or via indirect effects, e.g. related to GEM-derived metabolites (see [107] and references therein). Besides technical challenges relating to *in situ* efficacy (including survival and competition with indigenous microorganisms), such environmental concerns, and problems with solutions that were able to unequivocally alleviate them (e.g. conditional suicide mechanisms [107] have posed major hurdles to the commercialization of any GEM products for *in situ* use so far [56]. Upcoming approaches based on systems and synthetic biology could improve the issue of poor *in situ* efficiency of GEMs [109] and could thus make the release of GEMs for bioremediation applications more attractive. At the same time synthetic biology will allow to introduce increasingly complex genetic changes in organisms to generate new functions (and possibly to generate even entirely new organisms) which may, however, be also associated with the emergence of unexpected traits.

 Therefore new and possibly more efficient “firewall” mechanisms have been proposed in order to prevent genetic exchange of GEMs with, and potential long-term effects of GEMs on, native organisms in the environment [110, 111]. These mechanisms include non-natural nucleic polymers [xeno nucleic acids, (XNAs)] as genetic material (that cannot be read or duplicated by natural DNA or RNA polymerases), expanded or alternative genetic codes (that may encode both natural or non-natural amino acids), and cells dependent on xenobiotic chemicals (for recent review, see [112]). Interestingly, *in vitro* work lately demonstrated storage in and recovery of genetic information from various XNAs as well as the evolution of XNA aptamers, using enzymes that are able to convert DNA into XNA and vice versa. These enzymes had been generated by directed evolution (from an already mutated variant) of a replicative *Thermococcus* polymerase [113]. Though several point mutations were necessary to finally generate these converting enzymes [113], the findings may implicate the natural evolution of such enzyme activities, which would undermine a solely XNA-based firewall. This would make the integration in a single organism of additional xenobiotic “firewall levels” [112], like altered genetic codes, mandatory. However, even synthetic “xeno-organisms” that would show complete and durable genetic isolation from DNA-based life forms might still interact with native organisms on an ecological level, i.e. by changing habitats (e.g. via toxic metabolites), food webs or by competing out native populations. Such indirect effects could be relevant during the time period the synthetic organisms are supported with xenobiotic chemicals (that would be required for their growth), and/or after their death, e.g. due to putative long-lived toxic metabolites. Finally, XNAs can show increased stability compared to DNA and RNA; 1,5-anhydrohexitol nucleic acids (HNA) and locked nucleic acids (LNA), for instance, were described to be not susceptible to biological nucleases [114, 115]. In combination with the stable interaction of certain XNAs with DNA and RNA, which can experimentally and therapeutically be exploited to inhibit gene expression in various ways [116, 117], potential effects on native organisms related to increased XNA stability might be worth considering.

 As regards the environmental benefits to be gained from the industrial production of chemicals based on renewable sources, a reduction of energy needs and GHGs would indeed appear possible [118-120], though environmental impacts (e.g. carcinogens or eutrophication) of certain methods of producing biodegradable plastics may exceed those of fossil-based polymers [119]. Furthermore, large-scale production of bio-based chemicals (including fuels) may result in competition for land needed to grow food crops and may lead to GHG emissions from land-use change [120, 121] (see also biofuel debate below). 

### Energy

 Climate change mitigation and improved energy security could be the key assets of new generation of biofuels, if their production and use resulted in a net reduction of total GHG emissions. And if they were able to replace substantial proportions of fossil fuels, the burning of which meets over 80 % of energy needs worldwide – posing a substantial challenge in terms of the planet’s carbon cycle and global warming [122]. At the same time, they may reduce the need for land and competition for food as compared to first generation biofuels (see below).

 However, much as with the first generation biofuels, the new microbial “synthetic fuels” based on cane or corn sugar (see, (i)), may indirectly (i.e. by the need to grow corresponding plant feedstocks) end up as a factor driving developments such as the displacement of indigenous people, loss of biodiversity, or long-term net-GHG emissions as a result of land clearing and (direct or indirect) land-use change [22, 123-128]. Moreover, competition with food crops can become a factor with a negative impact on food security and food prices [129]. However, also lignocellulosic energy crops can generate net-GHG emissions, including those from fertilizer use and loss of organic soil carbon upon removal of crop residues [123, 125, 130], and may pose a threat to biodiversity (including effects by invasive energy crop species or fertilizer/pesticide pollution) [127, 131], depending on which type of land they are grown on. These issues may be reduced or avoided if plant feedstock can be grown on agriculturally-degraded or abandoned land with little or no fertilizer input [125-127, 131, 132]. Preventing food competition and displacement of people in affected countries, however, would require that “degraded” land were not needed for the livelihoods of indigenous populations.

 Though biofuels produced via photosynthetic microalgae may avoid these issues in principle, their benefits will depend on answers to crucial issues: namely the amount of water and energy (with associated GHG emissions) needed to grow and process algae, and the supply of CO_2_ and nutrients (fertilizer) [68, 133], the latter of which could bring algae-based strategies into conflict with food-crop production [68, 81]. Possible solutions include the use of wastewater (as a nutrient source), flue-gas CO_2_ and energy generation from spent algal biomass [81]. Furthermore, genetic engineering attempts to enhance light conversion efficiencies (see, e.g. [134]) and to generate direct synthesis pathways for biodiesel or hydrocarbons and/or their secretion from the algae [85, 86], may significantly reduce energy needs (avoiding energy-intensive processing and harvesting) [17].

 Thus important questions associated with the benefits of “synthetic” biofuels (see overview in Fig. **4C**), such as carbon neutrality/GHG emissions, loss of biodiversity and indeed human rights issues in poor countries (including rights of land holders or food security), may depend on feedstock, product, processing schemes and land use. Similarly, calculations of the proportion of fossil fuels that can be replaced by these new biofuels vary greatly depending on the development of yields, feedstock, products or land use [87, 135], lignocellulosic and algal biofuels possibly being able to replace the most substantial proportions [87, 136]. 

 Additional factors that can affect the equitable distribution of benefits and risks may include intellectual property rights and potential effects on the environment due to biosafety issues. The way patents for synthetic biology solutions are organized and applied may therefore influence the extent to which poor countries in the global south – which are likely to be the main areas for (plant-derived) biomass production [135] – have access to biofuel feedstock and technologies [137]. Furthermore, concerns have been raised that genetically engineered microorganisms such as microalgae could pose environmental risks if they escape, by becoming invasive and evolving rapidly [138].

## NEW BENEFITS, NEW RISKS, NEW GOVERNANCE?

 Synthetic genomics and synthetic biology, their potential implications on society as well as possible needs or options for their governance have been the topic of a number of reports by scientific organizations, policy advice institutions, civil society organizations and other players since the mid-2000s (see [139] and references therein). There appears to be broad consensus that it is paramount to maintain the trust of the public and policy regulators and that hype and exaggerated claims are counterproductive to developing regulatory models which respond to concerns of stakeholders and the public [139]. However, while some actors propose that current regulatory frameworks for recombinant DNA technology are still appropriate and the development of synthetic biology technologies and products should continue under these frameworks (see, e.g. [140]), a growing coalition of civil society organizations calls for a moratorium on the release and commercial use of synthetic organisms and the creation of new international regulations to govern the synthetic genomics/biology sector [141]. Others argue for significant public funding of ecological risks research on synthetic organisms and close cooperation between ecologists and synthetic biology researchers [142]. 

 As we have tried to outline in this article, there exists a wide spectrum of approaches currently connected to and discussed with the synthetic biology idea, ranging from simple genetic circuits to the generation of new metabolic pathways or synthetic viruses altered on the scale of the whole genome. In addition, these approaches can be part of different application schemes, including the production of chemicals from plant feedstocks in closed systems by GEMs, strategies that would require the release of GEMs (e.g. for bioremediation), or the use of genome synthesis to generate viral vaccines for use in humans. Many of these approaches and the organisms generated from them may not be clearly distinguishable from “traditional” genetic engineering and molecular biology approaches and their products – posing an additional issue on any proposed regulation (and indeed communication) on synthetic biology or “synthetic organisms”. The actual benefits and risks of those synthetic genomics/biology applications currently envisaged thus appear to depend on issues linked to different layers. 

 On the one hand, certain general aspects of application schemes appear to be decisive rather than synthetic biology-related issues. For example, the negative impacts on biodiversity or water/food security as a result of the large-scale planting of energy crops, intended for conversion to biofuels by genetically engineered/”synthetic” organisms, may be no different to those associated with any other energy-conversion technology using such biomass (such as Fischer-Tropsch synthesis, catalytic processes or pyrolysis schemes [69]). Nevertheless, a large increase in the scope of growing energy crops linked to an easier and more lucrative conversion to biofuels by “synthetic” organisms may make these issues more pressing. Similarly, broad patents and patent thickets – the number of which may increase as a result of synthetic biology approaches [103, 104] – already pose a challenge in the biopharmaceutical industry [143]. Curbing negative consequences of this kind on a global scale may require products or applications to be subjected to broadly applicable and effective environmental, socio-economic and ethical standards, irrespective of the exact nature of the underlying technical approach. In this way, negative impacts from bio-based chemicals or fuels could be mitigated by applying international sustainability and human rights standards to their production [22], and intellectual property issues could be addressed by international governmental organizations and industry within the framework of collaborative licensing models [137, 143]. 

 On the other hand, two threats more specifically associated with synthetic genomics/biology applications may emerge. (i) Large-scale custom DNA synthesis (and new genome-assembly techniques) combined with knowledge from functional genomics on pathogens might facilitate the generation and malicious use of new pathogens by both nation states and non-state actors (e.g. rogue individuals or terrorist groups). Various governance options have been suggested both within and outside the field, concerning screening procedures for DNA synthesis, its equipment and reagents, or ethical training of researchers (for recent overviews, see [20, 144]). Though much biotechnology knowledge and expertise has likely already proliferated globally [144], recent studies into various assessments on bioscience research and bioterrorism suggest that the attractiveness and feasibility of “synthetic” solutions for bioterrorist use may have to be reconsidered, especially when compared to available “low-tech” solutions [144, 145]. Still recent experiments, involving directed evolution and genetic-engineering, into the airborne transmission of the deadly bird-flu virus H5N1 in ferrets (a model to study influenza transmission in humans) [146, 147] have revigorated debates about conditions for publication of biosecurity-sensitive data [148]. (ii) As regards the other threat that is more specifically linked to the onset of synthetic biology, it may become more difficult, or even impossible, to assess the risks of extensively genetically modified or (putative) entirely “synthetic” future organisms, based on similarities with donor and recipient organisms. This issue may become more significant as the areas of synthetic genomics and synthetic biology progress and it remains to be seen whether synthetic biology-derived containment strategies (including xenobiotic mechanisms) can contribute to solve biosafety issues in future. Risk assessment may thus need to shift from prediction-based assessment to (more) real testing.

 In addition to these different layers of potential risks associated with synthetic genomics/biology applications, any assumption about the future benefits and risks needs to been seen in the light of the presumably low predictability of the exact nature of future innovations and application schemes from these emerging fields. Given this situation, effective governance should be informed by the most pluralistic expertise and perspectives available. Therefore, various actors may be involved by creating conditions that encourage and allow them to take on and evolve responsibilities regarding the development of scientific knowledge and the various levels of risks that may be associated with its use. Ideally, collaboration between governmental organizations, the academic and industrial synthetic genomics/biology communities, civil society organizations and the public would give rise to a safe yet dynamic web of mutual accountability and responsibility as the basis for a flexible and integrative governance approach to these integrative science fields.
